# Retraction: Immunotherapies against human bacterial and fungal infectious diseases: a review

**DOI:** 10.3389/fmed.2024.1518352

**Published:** 2024-11-01

**Authors:** Frontiers Editorial Office

The journal retracts the 14 April 2023 article cited above.

Following concerns regarding the originality of the article, an investigation was conducted in accordance with Frontiers' policies. It was found that the complaint was valid and that the article should be retracted, because of an unacceptable level of similarity to an article published by McCulloch, TR, Wells, TJ, and Souza-Fonseca-Guimaraes, F. Towards efficient immunotherapy for bacterial infection. Trends Microbiol. (2022) 30:158–69. 10.1016/j.tim.2021.05.005. In addition, copyright permissions were not obtained for the use of Table 1 and Figure 2, which have been removed from the published article.

This retraction was approved by the Chief Editors of Frontiers in Medicine and the Chief Executive Editor of Frontiers. The authors do not agree to this retraction.

